# The PRIDE database and related tools and resources in 2019: improving support for quantification data

**DOI:** 10.1093/nar/gky1106

**Published:** 2018-11-05

**Authors:** Yasset Perez-Riverol, Attila Csordas, Jingwen Bai, Manuel Bernal-Llinares, Suresh Hewapathirana, Deepti J Kundu, Avinash Inuganti, Johannes Griss, Gerhard Mayer, Martin Eisenacher, Enrique Pérez, Julian Uszkoreit, Julianus Pfeuffer, Timo Sachsenberg, Şule Yılmaz, Shivani Tiwary, Jürgen Cox, Enrique Audain, Mathias Walzer, Andrew F Jarnuczak, Tobias Ternent, Alvis Brazma, Juan Antonio Vizcaíno

**Affiliations:** 1 European Molecular Biology Laboratory, European Bioinformatics Institute (EMBL-EBI), Wellcome Trust Genome Campus, Hinxton, Cambridge CB10 1SD, UK; 2 Division of Immunology, Allergy and Infectious Diseases, Department of Dermatology, Medical University of Vienna, Vienna, 1090, Austria; 3 Ruhr University Bochum, Medical Faculty, Medizinisches Proteom-Center, D-44801 Bochum, Germany; 4 Applied Bioinformatics, Department for Computer Science, University of Tuebingen, Sand 14, 72076 Tuebingen, Germany; 5 Computational Systems Biochemistry, Max Planck Institute for Biochemistry, Martinsried, 82152, Germany; 6 Department of Congenital Heart Disease and Pediatric Cardiology, Universitätsklinikum Schleswig–Holstein Kiel, Kiel, 24105, Germany

## Abstract

The PRoteomics IDEntifications (PRIDE) database (https://www.ebi.ac.uk/pride/) is the world’s largest data repository of mass spectrometry-based proteomics data, and is one of the founding members of the global ProteomeXchange (PX) consortium. In this manuscript, we summarize the developments in PRIDE resources and related tools since the previous update manuscript was published in *Nucleic Acids Research* in 2016. In the last 3 years, public data sharing through PRIDE (as part of PX) has definitely become the norm in the field. In parallel, data re-use of public proteomics data has increased enormously, with multiple applications. We first describe the new architecture of PRIDE Archive, the archival component of PRIDE. PRIDE Archive and the related data submission framework have been further developed to support the increase in submitted data volumes and additional data types. A new scalable and fault tolerant storage backend, Application Programming Interface and web interface have been implemented, as a part of an ongoing process. Additionally, we emphasize the improved support for quantitative proteomics data through the mzTab format. At last, we outline key statistics on the current data contents and volume of downloads, and how PRIDE data are starting to be disseminated to added-value resources including Ensembl, UniProt and Expression Atlas.

## INTRODUCTION

High-throughput mass spectrometry (MS)-based proteomics approaches have matured significantly in recent years, becoming an increasingly used tool in biological research, sometimes together with other ‘omics’ approaches such as genomics and transcriptomics. Similarly, to what happened in those fields, in the last 15 years several public proteomics repositories and bioinformatics resources have been developed to support proteomics researchers. The PRoteomics IDEntifications (PRIDE) database (https://www.ebi.ac.uk/pride/) was set up in 2004 at the European Bioinformatics Institute (EMBL-EBI, Hinxton, Cambridge, UK) to enable public data deposition of MS proteomics data, providing access to the experimental data described in scientific publications (1). Since then, PRIDE (more concretely its archival component, PRIDE Archive) has evolved in parallel with the field becoming the largest proteomics data repository worldwide (2).

Although datasets coming from data-dependent acquisition (DDA) proteomics approaches represent by far the most abundant type of experiment, PRIDE Archive can store datasets coming from all main proteomics data workflows (including Data Independent Acquisition (DIA), MS imaging, and top down proteomics, among others). The mandatory data types to be included in each submitted dataset are the raw files (output files from the mass spectrometers) and the processed results (at least peptide/protein identification results, quantification information is optional). Therefore, each dataset in PRIDE Archive can contain heterogeneous data types such as peptide/protein identifications and quantification values, the mass spectra (peak lists and raw data), the searched sequence databases or spectral libraries, programming scripts and any other technical and/or biological metadata provided by the data submitters.

A key development led by PRIDE was the establishment of the ProteomeXchange (PX) consortium of MS proteomics resources (http://www.proteomexchange.org) (3), with the overall aim of standardizing data submission and dissemination of proteomics data worldwide. By September 2018, the following proteomics resources are also part of PX: PeptideAtlas and PASSEL (PeptideAtlaS SRM Experiment Library) (4,5), MassIVE (http://massive.ucsd.edu/), jPOSTrepo (6), iProx (http://www.iprox.org/) and Panorama Public (7).

PRIDE has four major aims: (i) support data deposition of proteomics experiments, and perform automatic and manual curation of the related experimental metadata; (ii) implement quality control pipelines and visualization components to enable the assessment of the data quality (8); (iii) promote and facilitate the re-use of public proteomics data; and at last, (iv) disseminate high-quality proteomics evidences to added-value resources, including Ensembl (9), UniProt (10) and Expression Atlas (11).

In order to facilitate the deposition, visualization and quality assessment of the data, the team has developed over the years a complete framework of open-source software, including stand-alone tools such as the PX Submission tool and PRIDE Inspector (12). In addition, the different PRIDE related data pipelines, REST web services (13) and the web interfaces (2) have been continuously refined. Furthermore, we have developed a number of open source software libraries in Java, including jmzML, jmzIdentML, jmzReader, jmzTab, ms-data-core-api (14) and the PIA (Protein Inference Algorithms) toolbox (15,16) (https://github.com/PRIDE-Utilities), to support handling (e.g. read and writing) of the most popular proteomics data standard formats (e.g. mzML, mzIdentML, mzTab) developed by the Proteomics Standards Initiative (PSI) (17). In addition to all the PX resources mentioned above, there are additional proteomics databases and resources available providing protein expression information, most notably the Global Proteome Machine Database (GPMDB) (18), the CPTAC (Clinical Proteomic Tumor Analysis Consortium) data portal (19) and ProteomicsDB (20).

In this manuscript, we will summarize the main PRIDE related developments in the last three years, since the previous *Nucleic Acids Research* database update manuscript was published (2). We will discuss PRIDE Archive in more detail but will also provide updated information about the PRIDE related tools and other ongoing activities.

## CURRENT STATUS OF PRIDE ARCHIVE AND RELATED TOOLS

**Figure 1.**
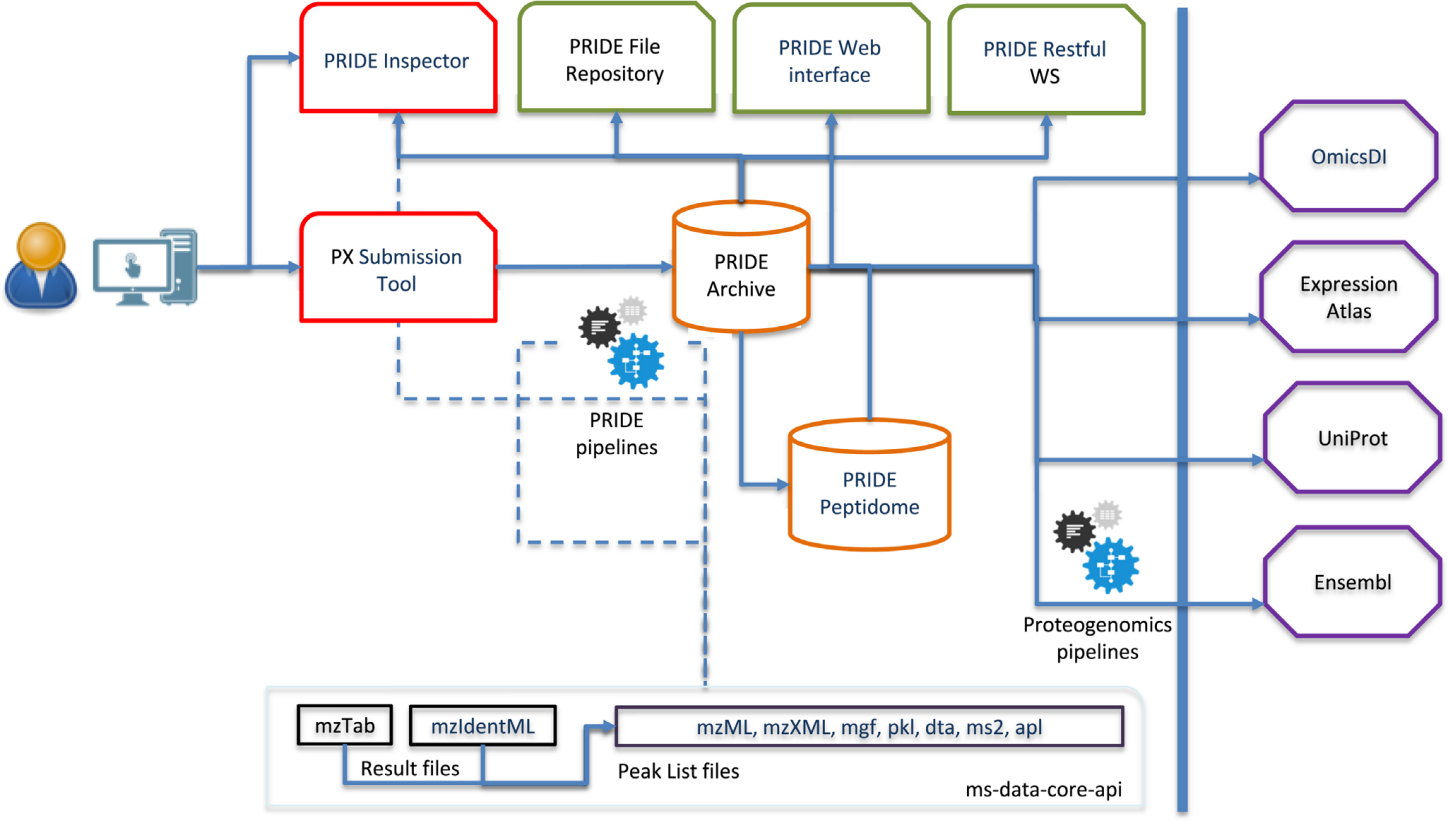
Overview of the PRIDE ecosystem, including the resources (PRIDE Archive and PRIDE Peptidome, in orange), tools (PRIDE Inspector and PX Submission Tool, in red), software libraries (in black), web interface and API (in green) and the external resources where PRIDE data are disseminated to (in purple).

Original submitted datasets by scientists are stored in PRIDE Archive (http://www.ebi.ac.uk/pride/archive/). All datasets remain private (password protected) by default and are only made publicly available after the related manuscript has been accepted, or when PRIDE is notified to do so by the original submitter. Data in PRIDE Archive can be searched and accessed in four different ways: (i) the web interface, providing a general overview of each dataset; (ii) the PRIDE Inspector tool (12), which can be used for downloading the submitted data files and to visualize spectrum, peptide and protein information in open formats, including several PSI standards; (iii) the Restful web service (https://www.ebi.ac.uk/pride/ws/archive/) (21); and (iv) a file repository, where both the FTP and Aspera (https://asperasoft.com/) file transfer protocols can be used to access the files. In addition, all public datasets in PRIDE Archive are available through OmicsDI (https://www.omicsdi.org/), an EMBL-EBI resource which integrates public datasets coming from different omics technologies (22). Figure 1 provides an overview of the PRIDE ecosystem, including the most relevant tools, software libraries and the data dissemination into other resources.

### New PRIDE Archive infrastructure: scaling-up a resource for present-day proteomics experiments

The number of datasets submitted to PRIDE has grown very significantly in recent years, in parallel with the size of the experiments, e.g. the number of samples, biological/technical replicates and evidences—mass spectra, Peptide Spectrum Matches (PSMs), peptides and proteins. Two different factors, scalability and reliability (fault-tolerance), have guided the development of the new PRIDE Archive distributed architecture (Supplementary Note 1). Every storage item (e.g. MongoDB, Solr Indexes) is now deployed in two EMBL-EBI datacenters as shard distributed clusters. This new architecture ensures that if one datacenter is not accessible (due to e.g. technical maintenance), PRIDE Archive is still accessible.

### Data submission process: improved support for quantification results

The data submission process has not substantially changed because the overall PX submission guidelines have remained stable (23). An updated web tutorial explaining the process is available at http://www.ebi.ac.uk/training/online/course/proteomexchange-submissions-pride. The main addition is the support for the standardized tab-delimited mzTab format (24) to perform ‘Complete’ submissions (those where peptide/protein identifications and, thanks to this ongoing development, also the corresponding quantitative information, can be parsed by the repository, made accessible in the database and linked to the originating mass spectra). Therefore, support for mzTab has enabled the deposition of quantitative data into PRIDE Archive for the first time in a standard format that is supported for ‘Complete’ submissions (Supplementary Note 2). By October 2018, the Mascot (25) search engine (e.g. https://www.ebi.ac.uk/pride/archive/projects/PXD009079), the OpenMS framework (26) (e.g. https://www.ebi.ac.uk/pride/archive/projects/PXD010981) and MaxQuant (27) (e.g. https://www.ebi.ac.uk/pride/archive/projects/PXD011194) enable natively the export of quantitative results into mzTab. In order to keep improving the support for quantification data, we aim to promote the implementation of mzTab in other popular software tools such as Proteome Discoverer (*ThermoFisher Scientific*).

The mzIdentML format remains as the mainstream format for ‘Complete’ submissions and is increasingly supported by search engines and tools (14). In case mzTab and/or mzIdentML are not yet supported by the user’s software of choice, the alternative is to perform a ‘Partial’ submission, which is also the current alternative for data workflows such as DIA, top-down and MS imaging. In parallel with the ongoing developments in PSI data standard formats, all PRIDE-related software libraries (https://github.com/PRIDE-Utilities) have been continuously developed, making data handling and submission a much more robust process. In this context, we will continue extending our libraries (ms-data-core-api and jmzIdentML) to support the new features included in mzIdentML version 1.2 such as MS/MS cross-linking and proteogenomics approaches.

### The PX submission tool

The PX Submission tool (3) (available at https://github.com/proteomexchange/px-submission-tool) is a stand-alone tool used by most PRIDE submitters to perform data submissions. Some of the recent refinements done in the tool are: (i) the integration of the new OLS (Ontology Lookup Service) Client and OLS Dialog libraries (28), supporting the new version of the OLS, used to annotate datasets using controlled vocabulary terms; and (ii) the addition of a direct feedback system for users to report how the data submission went.

### PRIDE Inspector toolsuite: reviewing datasets before and after submission to PRIDE Archive

The PRIDE Inspector tool (12) (available at https://github.com/PRIDE-Toolsuite/pride-inspector) was developed to enable researchers to visualize and perform an initial quality assessment of the data both before and after data submissions are performed, once the dataset becomes public. PRIDE Inspector supports several different experimental open output files, ranging from mass spectra (mzML, mzXML and the most popular peak lists formats such as mgf, dta, ms2, pkl and apl), identification results (mzIdentML, mzTab), to quantification data (mzTab). Some refinements have been implemented in the tool and in the underlying software libraries over these last years. The main new feature added recently is the support for reviewers to download private datasets using the much faster Aspera file transfer protocol. This key functionality to facilitate the review process is not available via the PRIDE Archive web interface at present.

### PRIDE web interface and restful API: retrieving public proteomics data

**Figure 2.**
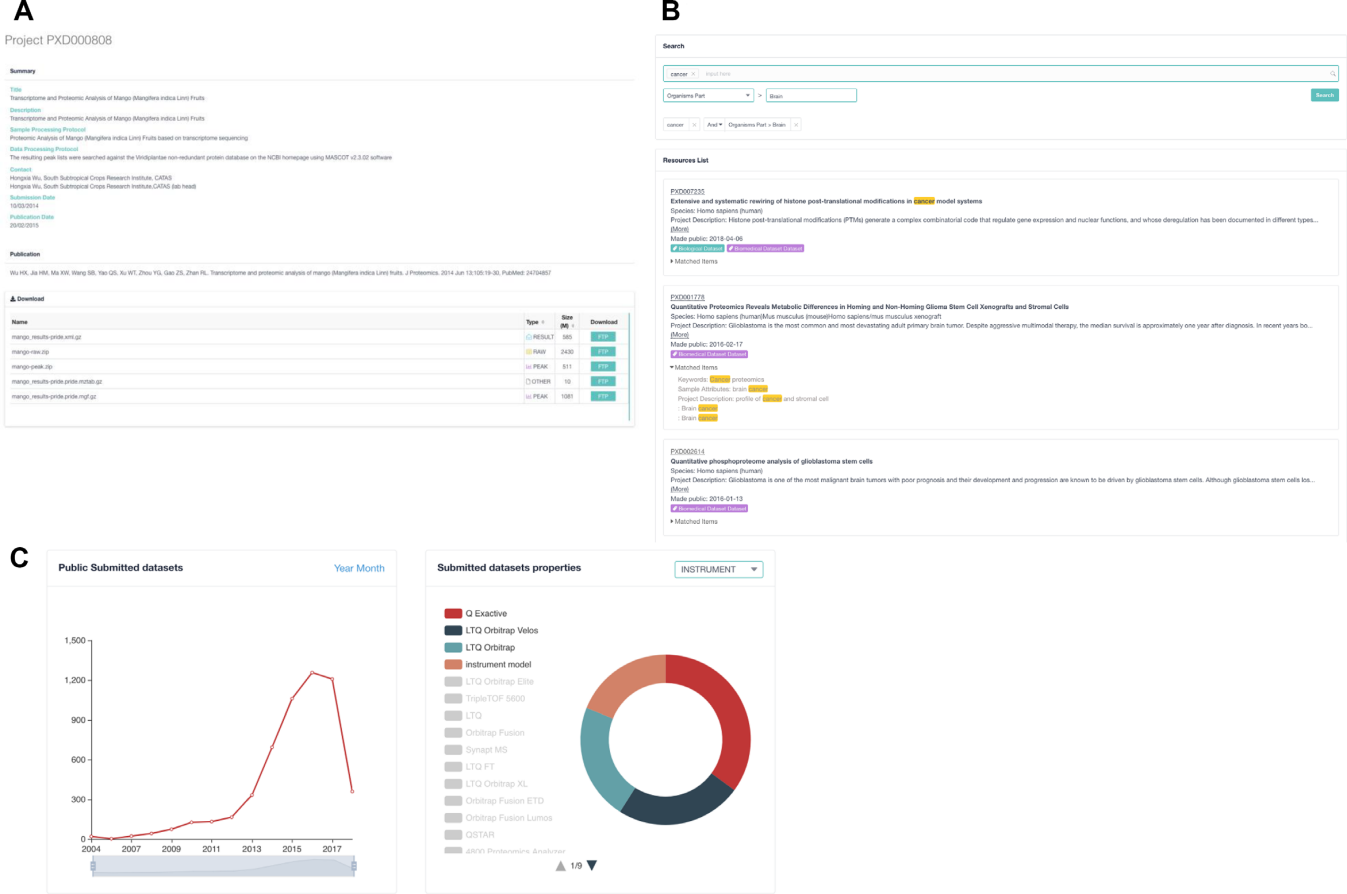
Screenshots of the new PRIDE Archive web interface. (**A**) The project (dataset) page provides a general overview of every submitted dataset. (**B**) The PRIDE Archive search page, where it is possible for users to query PRIDE Archive using keywords and additional properties such as species, tissues and instruments, among others. (**C**) Real-time statistics (including number of submitted datasets per month, number of submitted datasets per instrument type, etc.) are now provided.

The PRIDE web interface and Restful API (13) can be used to retrieve and visualize the data corresponding to all PRIDE datasets. The new PRIDE web interface (Figure 2) provides a powerful mechanism to search and/or filter by several types of metadata information, such as sample details (e.g. species, tissue, cell type, etc.), instrumentation (mass spectrometer), keywords and other provided annotations (Supplementary Note 3). Using the API, it is possible to programmatically query for and retrieve peptide and protein identifications, dataset and assay specific metadata, and all the originally submitted files. Both components are currently under development and new functionalities are being implemented such as suggestions for similar datasets, auto-complete search capabilities and live data content statistics (Figure 2).

### PRIDE Peptidome: high-quality peptide evidence from PRIDE Archive

The PSMs reported in PRIDE Archive are quality-filtered using a spectrum clustering approach (29). All the identified spectra coming from the public experiments in PRIDE Archive were clustered using the second iteration of the PRIDE Cluster algorithm, called *spectra-cluster* (https://github.com/spectra-cluster) (30). The results of the clustering process are made available through the peptide centric PRIDE Peptidome resource (formally known as PRIDE Cluster, http://www.ebi.ac.uk/pride/cluster/), which has a completely new web interface, in line with the new PRIDE Archive one. The corresponding spectral libraries and spectral archives (containing only unidentified spectra) are made available at https://www.ebi.ac.uk/pride/cluster/#/libraries and at https://www.ebi.ac.uk/pride/cluster/#/results.

## PRIDE ARCHIVE DATA CONTENT STATISTICS

**Figure 3.**
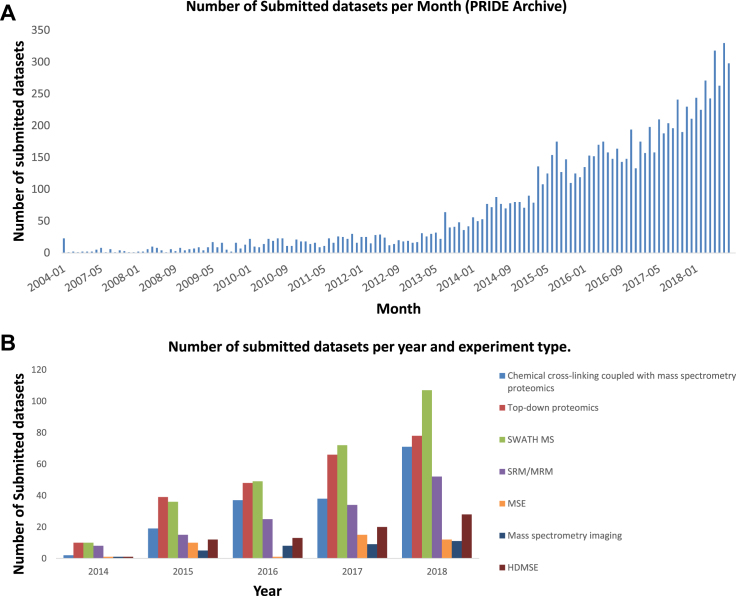
(**A**) Number of submitted datasets to PRIDE per month (from beginning of 2004 till September 2018). (**B**) Number of submitted datasets per experimental approach per year (from 2014 till September 2018).

By 1 September 2018, PRIDE Archive contained 10100 datasets (compared to 3336 datasets on September 2015), of which roughly 19% are ‘Complete’ (1975 datasets), 72% are ‘Partial’ (7295) and the remaining 9% (830) correspond to old ‘legacy’ datasets submitted before the PX data workflow was implemented. Figure 3A shows the evolution in the number of submitted datasets per month. By September 2018, an average of 274 datasets were submitted per month during 2018, amounting to more than 2-fold when compared with 3 years ago. The landmark dataset PXD010000 was submitted on 1 June 2018. These figures correspond to all datasets including private ones (non-released, password protected). By 1 September 2018, 56% (5719) of the datasets were publicly available. Interestingly, the number of submitted datasets generated using experimental approaches other than DDA is growing (Figure 3B). By September 2018, the number of datasets classified as DDA in PRIDE was 91%, while 26% was classified as other types (Figure 3B). The number of DIA and selected reaction monitoring (SRM) datasets are indeed the most abundant ones behind DDA datasets.

**Figure 4.**
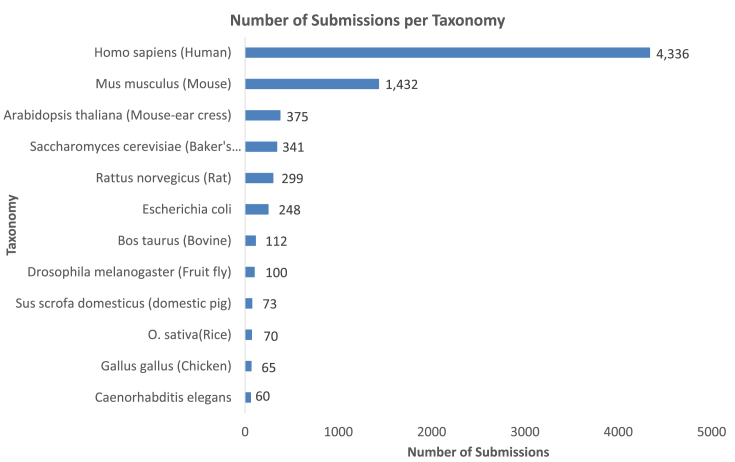
Number of submitted datasets to PRIDE Archive per taxonomy identifier.

The most represented species (including both public and private datasets) are human (4335 datasets) and some of the main model organisms, most notably mouse (1432), *Arabidopsis thaliana* (375), *Saccharomyces cerevisiae* (341), rat (300), *Escherichia coli* (247), cow (112), *Drosophila melanogaster* (101), chicken (65), rice (70) and soybean (49). Overall, datasets coming from more than 1840 different taxonomy identifiers are stored in PRIDE Archive (Figure 4). These statistics represent in our view a fair reflection of the current guidelines for mandatory data deposition developed by many funding agencies and some scientific journals. At the time of writing, the Wellcome Trust, BBSRC, MRC and the NIH, among other funders, mandate or strongly encourage open access to research data including proteomics. Additionally, two of the most prominent proteomics journals (*Molecular and Cellular Proteomics* and *Journal of Proteome Research*) and journals from the *Nature* group now mandate submission of at least the raw data supporting each proteomics publication. Other journals already recommend or strongly recommend data submission (e.g. *Proteomics* (Wiley), *Journal of Proteomics* (Elsevier), *PLOS* journals, etc.). The evolution in the percentage of research articles supported by PRIDE datasets (in three different proteomics journals: *Molecular and Cellular Proteomics, Journal of Proteome Research* and *Proteomics*) is explained in Supplementary Note 4. At last, in this context, it is important to highlight that the Human Proteome Project has developed formal guidelines mandating data submission for all generated datasets (31).

## DATA RE-USE OF PUBLIC PRIDE DATASETS

Proteomics researchers are increasingly re-using public data available in PRIDE (and other resources) for a broad range of purposes. We came up with four categories of public proteomics data re-use: (i) *use*, (ii) *re-use*, (iii) *reprocess* and (iv) *repurpose* (32). A simple example of the direct *use* of data are given by connecting information between proteomics data resources and other resources such as UniProt and Ensembl (10). In the case of *re-use*, public data are re-used in novel experiments with the potential of generating new knowledge. Data from a large number of independent datasets can be analyzed or re-used in combination (a so-called *meta*-analysis study), to extract new knowledge not accessible from any individual dataset. In the case of *reprocess*, public datasets are re-analyzed to provide an updated or integrated view on the results, as protein sequence databases and software tools evolve. At last, *repurposing* includes all those cases where the data are considered in a context that is different to that of the original experiment. Two popular applications are proteogenomics approaches (for human and the main model organisms, e.g. (33,34)), and the discovery of novel PTMs (Post-Translational Modifications). Recent reviews in re-use of public proteomics data are available (32,35).

**Figure 5.**
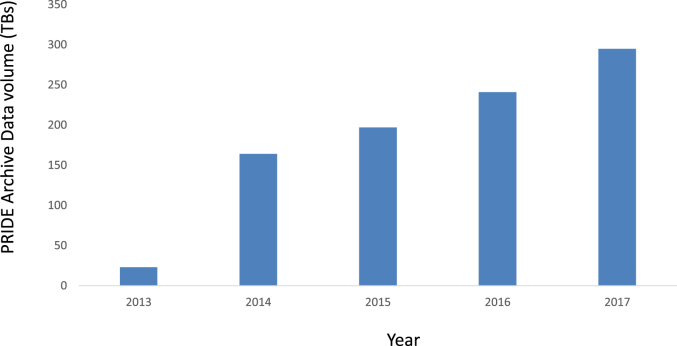
Data volume (in terabytes) downloaded from PRIDE Archive per year.

To corroborate the increase in data re-use, Figure 5 shows the increase in the volume of PRIDE data downloads per year, reaching 296 TBs during 2017. In addition, using the previously mentioned resource OmicsDI, it is now possible to trace the number of re-analyses of PRIDE datasets performed by PeptideAtlas and GPMDB and the number of direct citations of PRIDE datasets in the literature (BioRxiv: https://doi.org/10.1101/282517). By September 2018, 293 datasets had been re-analyzed and 381 dataset identifiers had been cited directly in the literature.

### PRIDE Proteogenomics: representing peptide sequences into Ensembl using ‘TrackHubs’

**Figure 6.**
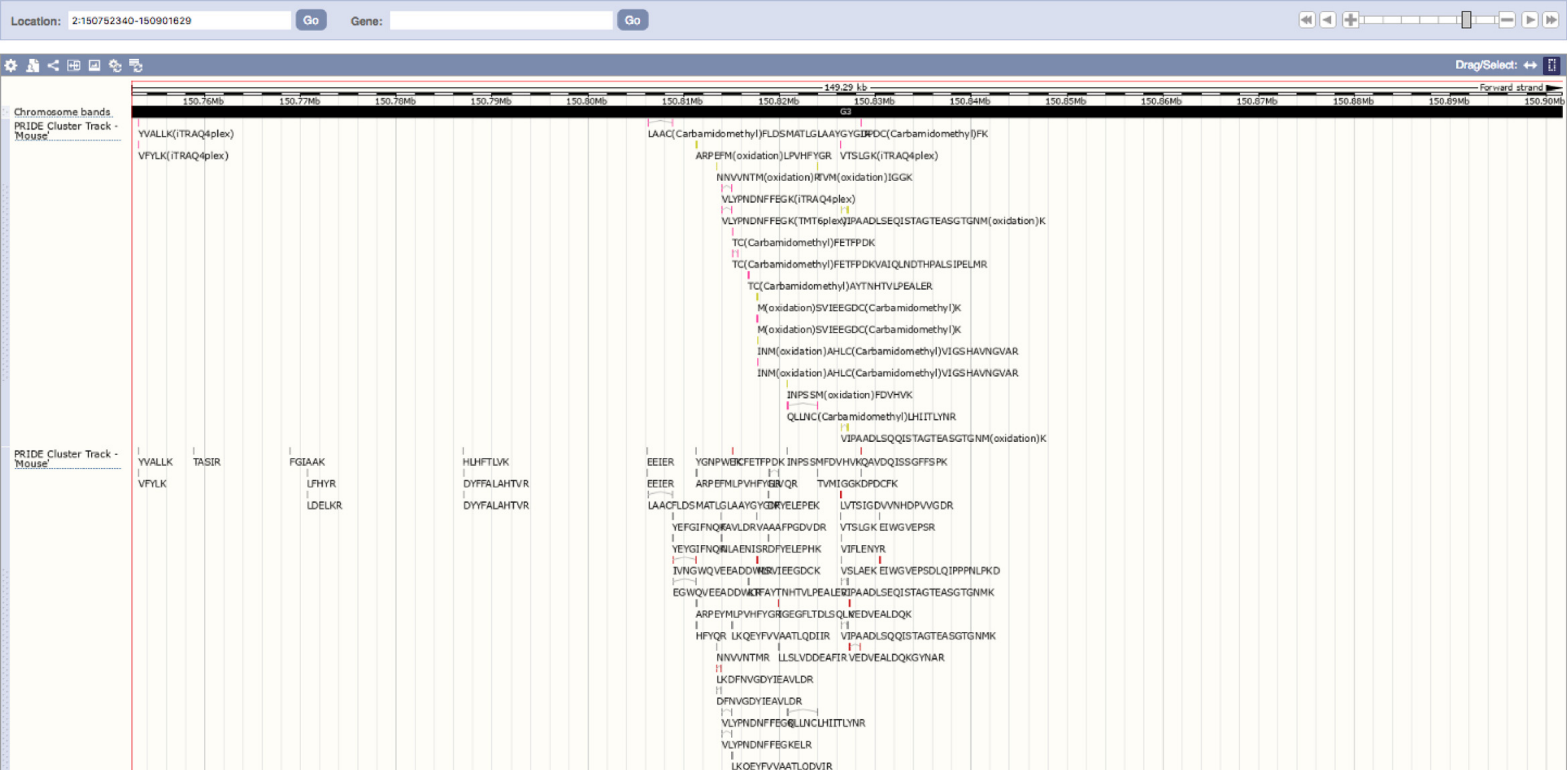
Screenshot of the Ensembl genome browser showing the visualization of peptide evidence as ‘TrackHubs’ coming from PRIDE Archive and PRIDE Cluster (now PRIDE Peptidome). All peptides shown come from mouse data (GRCm38).

The PRIDE and Ensembl teams have been working together to improve the integration of proteomics data in a genome context. Peptide evidence from ‘complete’ public datasets in PRIDE Archive are first quality-filtered (at a 1% peptide false discovery rate) using a framework that uses PIA (15). Reliable peptide sequences (including PTMs) are mapped to the corresponding genomic coordinates from a given Ensembl release using the PoGo tool (36). The resulting data for each individual dataset is then combined and made available through the Ensembl ‘TrackHub’ registry, using the popular BED format. In addition to individual datasets, PRIDE Cluster data (now re-named to PRIDE Peptidome) is also made available as independent 'TrackHubs'. At the time of writing, 184 PRIDE public datasets have been already made available in the Ensembl ‘TrackHub’ registry (https://www.trackhubregistry.org/): 163 human, 15 from *Mus musculus*, 4 from *Rattus norvegicus* and 2 from *Bos taurus*. The ‘TrackHubs’ can be searched in the ‘TrackHub’ registry by project identifier, taxonomy and/or specific keywords available in the description of the corresponding PRIDE dataset. As a key point, ‘TrackHubs’ can be loaded and visualized in the Ensembl web interface together with other genomic features (Figure 6). More than 4 million peptide sequences (1.2 million of them containing PTMs) have been mapped to the human genome (GRCh38). We are working in including data coming from other model organisms. It is very important to highlight that the developed framework supports the other two major genome browsers: The UCSC Genome Browser and IGV (Integrative Genomics Viewer). All data can be downloaded from http://ftp.pride.ebi.ac.uk/pride/data/proteogenomics/latest/archive/, for downstream analysis.

### Moving data into Expression Atlas: re-analysis of quantitative datasets

At the time of writing, 15 quantitative proteomics datasets have already been integrated into the Expression Atlas, an EMBL-EBI added value database that provides information about gene and protein expression in different species and contexts (11). All PRIDE integrated proteomics datasets were manually curated and re-analyzed using a MaxQuant based pipeline. By September 2018, five mouse datasets (showcasing a complete proteome, e.g. https://www.ebi.ac.uk/gxa/experiments/E-PROT-16/Results), six datasets coming from cancer cell lines (showcasing the integration between proteomics and transcriptomics data) and four datasets coming from clinical tumor samples had already been integrated in Expression Atlas. From Expression Atlas, in the near future, we plan that relevant quantitative proteomics data will be disseminated into the Open Targets platform (37).

## DISCUSSION AND FUTURE PLANS

Thanks, among other efforts, to the success of PRIDE (and of the PX consortium as a whole), the proteomics community is now widely embracing open data policies, an opposite scenario to the situation just a few years ago. In parallel, public proteomics data are being increasingly re-used, with multiple applications. We next outline some of the main working areas for PRIDE in the near future.

First of all, a key aspect is to improve the annotation of the datasets. The current requirements were set up in 2011 (minor updates in 2013), during the establishment of PX, reflecting the discussions at the time, involving many key stakeholders in the field. The main priority was to make data sharing popular. Once this has been achieved, it is now the right time to ‘raise the bar’. At the time of writing, a novel annotation system is under development (Supplementary Note 2). The aim is to improve the capture of the experimental design information and technical metadata (e.g. search parameters and relevant information contained in the raw files) (28,38). The improvement in annotation is also required to facilitate further data re-use for third parties. Another key aspect in making data re-use easier is to bring the analysis tools closer to the data, as datasets keep increasing in size.

We are already working in developing open and reproducible data analysis pipelines of different flavours of proteomics workflows (e.g. DDA, DIA, proteogenomics). The main rationale is to make possible the use of that software in cloud infrastructures (using the EMBL-EBI cloud as the starting point), so that in the future the pipelines can be used by the community in the cloud using software container technologies (39,40). In addition, we aim to increasingly perform internal data re-use (including data re-processing) and disseminate high-quality proteomics data from PRIDE into the already mentioned added-value resources (Ensembl, UniProt and Expression Atlas), among others. At present, identified proteins in PRIDE ‘Complete’ datasets are cross-referenced in the corresponding UniProt entries (e.g. https://www.uniprot.org/uniprot/Q12181) and ‘TrackHubs’ are published for some ‘Complete’ datasets in Ensembl. We plan to enable a more detailed annotation of UniProt and Ensembl entries using proteomics evidence coming from PRIDE, focusing on PTMs, sequence variants and quantitative expression information.

To support this, integration of re-analyzed datasets and the corresponding results in the PRIDE Archive infrastructure needs to be properly supported. Another highly relevant topic for the coming years is the management of clinical proteomics data, and whether they should be considered as patient identifiable or not. This topic has recently gained more relevance after the introduction of the GDPR (General Data Protection Regulation) guidelines by the European Union and we plan to discuss it further in the context of the ELIXIR activities (https://www.elixir-europe.org/). In this context, it is important to highlight that in 2017, PRIDE was named an ELIXIR core data resource (https://www.elixir-europe.org/platforms/data/core-data-resources), joining those biological databases considered to be essential for the scientific community, highlighting the need to make them sustainable in the long term (41).

To finalize, we invite interested parties in PRIDE related developments to follow the PRIDE Twitter account (@pride_ebi). For regular announcements of all the new publicly available datasets, users can follow the PX Twitter account (@proteomexchange).

## Supplementary Material

Supplementary Data
