# Supplementary material for: The PRIDE database and related tools and resources in 2019: improving support for quantification data
Source: Nucleic Acids Res. 2018 Nov 5;47(Database issue):D442–50. doi: 10.1093/nar/gky1106 (PMC6323896; doi:10.1093/nar/gky1106)
Supplement: Supplementary Data [file gky1106_supplemental_files.docx]

**Supplementary Notes: “The PRIDE database and related tools and resources in 2019: Improving support for quantification data”.**

**Supplementary Note 1: New PRIDE Archive Architecture**

The new PRIDE Architecture includes a distributed storage system with two data centres that enable the replication and scalability of the databases (**Supplementary Figure 1**). With this new architecture, PRIDE Archive can: i) receive and manage more efficiently the increasing number of submissions per day; ii) eliminate the possibility of down-time because data will be served by two datacentres (if one datacentre is down, the other datacentre can still serve the data); and iii) provide improved scalability. The multiple-shard clusters (Solr and MongoDB) can serve more data in a faster manner when compared with the previous architecture based on a single machine approach.

**
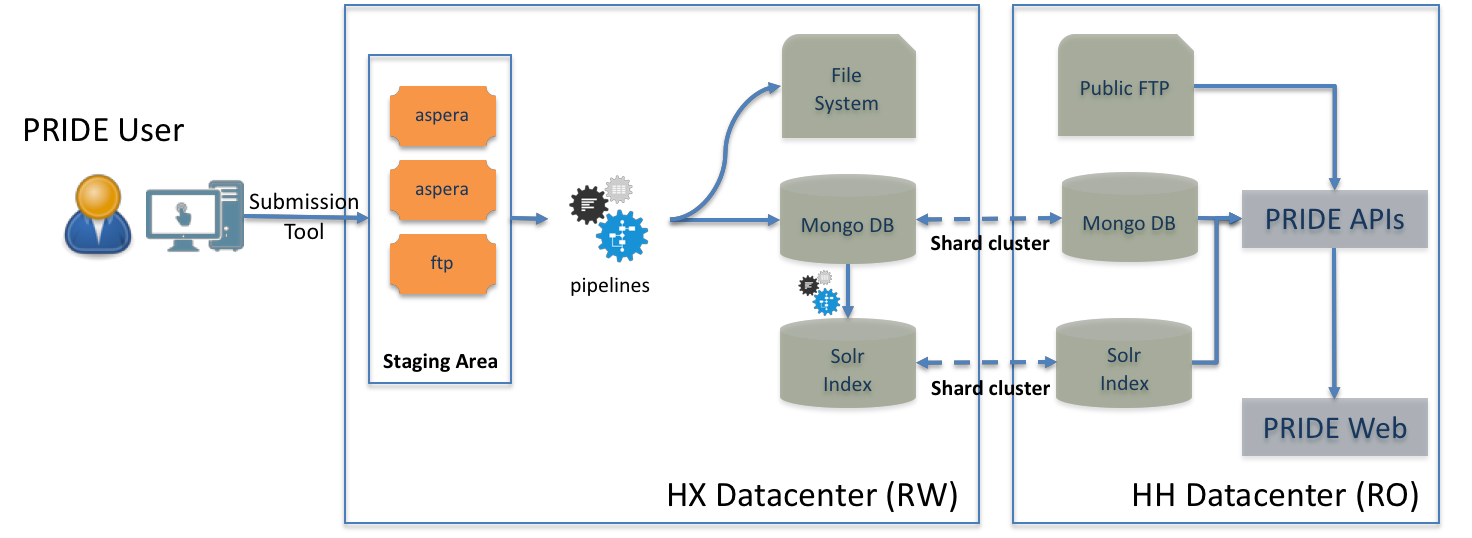
**

**Supplementary Figure 1**: Scheme of the new PRIDE Archive Architecture.

**Supplementary Node 2: PRIDE RoadMap for supporting quantitative proteomics results**

Since 2014 PRIDE has been working toward providing an improved support on public data deposition for quantitative data (**Supplementary Figure 2**). In 2014, the HUPO-PSI (Proteomics Standards Initiative) community (led by PRIDE) developed the mzTab standard file format, which can be used to capture quantitative information at the peptide and protein level (1). This new file format triggered the development of new Java libraries (e.g. jmzTab and ms-data-core-api) and a new version of the PRIDE Inspector Toolsuite, which was the first tool able to visualize mzTab files (2,3). During 2017, the team updated all the internal pipelines (submission, validation and publication) to support ‘Complete’ submissions based on the mzTab file format. It is important to highlight that the PRIDE pipelines can validate every single result file included in ‘Complete’ submissions. As a key point, the pipeline checks that for every PSM the corresponding spectrum is present and properly linked. In 2018, the PRIDE team and other members of the HUPO-PSI community have been working together with software developers of different analysis tools to implement native export to mzTab. As a result, during 2018, three major quantitative analysis frameworks have implemented mzTab export: functionality: Mascot (4), MaxQuant (5) and OpenMS (6).

The next major developments in this area will be focused on improving the experimental design and metadata capture of quantitative experiments (**Supplementary Figure 2b**). In 2017, we updated the PRIDE software to interact with the ontology lookup service (OLS) (7). OLS is a central component to enable the annotation of the experimental design using ontology terms. At the time or writing, the PRIDE team is building a lightweight annotation system for PRIDE curators and external users to be able to annotate PRIDE datasets after the initial submission was performed. In addition, automatic annotation pipelines are being implemented to facilitate the annotation of technical metadata, including e.g. search parameters and information contained in the MS runs (raw files) (8).

**
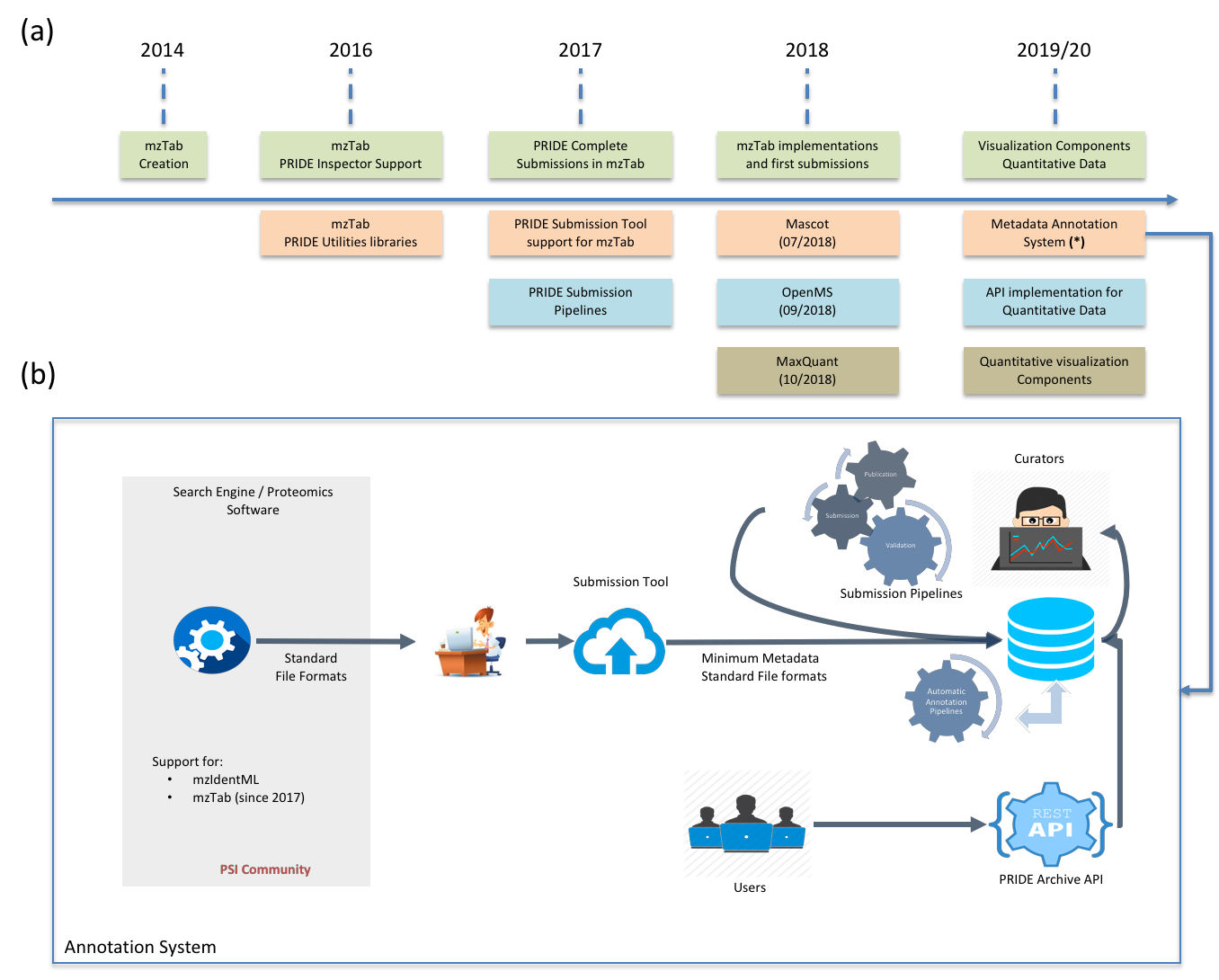
**

**Supplementary Figure 2:** PRIDE roadmap for supporting quantitative proteomics datasets (a) PRIDE quantitative support for ‘complete’ submissions is based in the mzTab file format. (b) A complete annotation system to capture better the experimental design and technical metadata is currently under development.

**Supplementary Note 3: PRIDE Restful API 2.0**

The new PRIDE API (<http://wwwdev.ebi.ac.uk/pride/ws/archive/swagger-ui.html>, the 1.0 release version is expected to be available by December 2018 in <http://www.ebi.ac.uk/pride/ws/archive/>, so this need to be taken into account to construct all the URLs included in this section) provides a better functionality for querying PRIDE data. A new external documentation page has been created (<http://wwwdev.ebi.ac.uk/pride/ws/archive/docs/api-guide.html>) to explain every functionality and end-point in the Restful API. Some of the most important novel functionalities are the following:

- New filtering methods at the project and file level. For example, the query <http://wwwdev.ebi.ac.uk/pride/ws/archive/files?filter=publicationDate%3Drange%3D%5B2006-01-01%20TO%202015-12-31%5D&pageSize=100> will provide a list of all files submitted between 2006 and 2015. Additionally, this query can be combined with the file type (e.g. raw or Result files), etc.
- Pagination and Hypermedia functionality: A new pagination system has been implemented to avoid performance deadlock. Additionally, users can navigate the API using hypermedia links (e.g. [http://wwwdev.ebi.ac.uk/pride/ws/archive/docs/api-guide.html#](http://wwwdev.ebi.ac.uk/pride/ws/archive/docs/api-guide.html)). This new feature makes also the API self-discoverable.
- Additional entry points: New end-points to retrieve the specific data (e.g. files) directly by using filtering properties have been implemented.

**Supplementary Note 4: Coverage of PRIDE datasets in the main proteomics journals**

One way to get insights into the changing willingness to share proteomics data in the community is to analyse the number of articles that are supported by PRIDE datasets. To estimate this, we have collected and manually curated PRIDE dataset mentioned in published relevant proteomics papers in three of the most prominent proteomics scientific journals: *Molecular Cellular Proteomics* (MCP - <http://www.mcponline.org/>), *Proteomics* (<https://onlinelibrary.wiley.com/journal/16159861>) and *Journal of Proteome Research* (JPR - <https://pubs.acs.org/journal/jprobs>). We did it in four different arbitrary time points of 2 months each:

- May and April 2012: before the launch of ProteomeXchange (PX)

- December 2014 and January 15: 2.5 years after launching PX.

- September and October 2016: 4 years after PX launch.

- June and July 2018: 6 years after PX launch.

**Supplementary Figure 3** below summarises the data coming from the four different time points studied. In the case of MCP, the coverage went up from 0 (in 2012) to ~70% in 2016 and it has stayed roughly at the same level (66%) in 2018. For JPR it went from the almost non-existent 3% in 2012 to 18% in 2014/2015. Then it almost doubled, reaching 34% in 2016, and it still has grown significantly since reaching 56% in 2018. The journal *Proteomics* already had a sizeable percentage of its papers backed by PRIDE datasets in 2012 (25%) and that number went up to ~40% by 2014/2015. However, in 2016 the percentage went down to 27% and this number further reduced to 17% in 2018. However, it is important to note that the journal *Proteomics* only accounted for a small percentage of all the papers published by the three journals (for instance in 2018 this meant only 15 studies out of the 106 examined). This fact is also implicit in the figures comprising the three journals combined, reaching a percentage of ~54% in 2018.


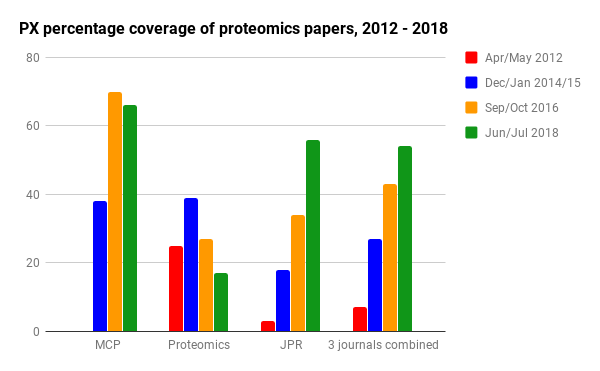


**Supplementary Figure 3**: Histogram summarising the percentage of papers backed by PX datasets in the four studied data points.

**References**

1. Griss, J., Jones, A.R., Sachsenberg, T., Walzer, M., Gatto, L., Hartler, J., Thallinger, G.G., Salek, R.M., Steinbeck, C., Neuhauser, N. *et al.* (2014) The mzTab data exchange format: communicating mass-spectrometry-based proteomics and metabolomics experimental results to a wider audience. *Mol Cell Proteomics*, **13**, 2765-2775.

2. Perez-Riverol, Y., Uszkoreit, J., Sanchez, A., Ternent, T., Del Toro, N., Hermjakob, H., Vizcaino, J.A. and Wang, R. (2015) ms-data-core-api: an open-source, metadata-oriented library for computational proteomics. *Bioinformatics*, **31**, 2903-2905.

3. Perez-Riverol, Y., Xu, Q.W., Wang, R., Uszkoreit, J., Griss, J., Sanchez, A., Reisinger, F., Csordas, A., Ternent, T., Del-Toro, N. *et al.* (2016) PRIDE Inspector Toolsuite: Moving Toward a Universal Visualization Tool for Proteomics Data Standard Formats and Quality Assessment of ProteomeXchange Datasets. *Mol Cell Proteomics*, **15**, 305-317.

4. Perkins, D.N., Pappin, D.J., Creasy, D.M. and Cottrell, J.S. (1999) Probability‐based protein identification by searching sequence databases using mass spectrometry data. *ELECTROPHORESIS: An International Journal*, **20**, 3551-3567.

5. Tyanova, S., Temu, T. and Cox, J. (2016) The MaxQuant computational platform for mass spectrometry-based shotgun proteomics. *Nat Protoc*, **11**, 2301-2319.

6. Pfeuffer, J., Sachsenberg, T., Alka, O., Walzer, M., Fillbrunn, A., Nilse, L., Schilling, O., Reinert, K. and Kohlbacher, O. (2017) OpenMS–A platform for reproducible analysis of mass spectrometry data. *Journal of biotechnology*, **261**, 142-148.

7. Perez-Riverol, Y., Ternent, T., Koch, M., Barsnes, H., Vrousgou, O., Jupp, S. and Vizcaino, J.A. (2017) OLS Client and OLS Dialog: Open Source Tools to Annotate Public Omics Datasets. *Proteomics*, **17**.

8. Hulstaert, N., Reisinger, F., Rameseder, J., Barsnes, H., Vizcaino, J.A. and Martens, L. (2013) Pride-asap: automatic fragment ion annotation of identified PRIDE spectra. *J Proteomics*, **95**, 89-92.
